# Clonal and serotype dynamics of serogroup 6 isolates causing invasive pneumococcal disease in Portugal: 1999-2012

**DOI:** 10.1371/journal.pone.0170354

**Published:** 2017-02-02

**Authors:** Jorge Diamantino-Miranda, Sandra Isabel Aguiar, João André Carriço, José Melo-Cristino, Mário Ramirez

**Affiliations:** Instituto de Medicina Molecular, Faculdade de Medicina, Universidade de Lisboa, Av. Prof. Egas Moniz, Lisboa, Portugal; Rockefeller University, UNITED STATES

## Abstract

Although serogroup 6 was among the first to be recognized among *Streptococcus pneumoniae*, several new serotypes were identified since the introduction of pneumococcal conjugate vaccines (PCVs). A decrease of the 6B-2 variant among invasive pneumococcal disease (IPD), but not 6B-1, was noted post conjugate vaccine introduction, underpinned by a decrease of CC273 isolates. Serotype 6C was associated with adult IPD and increased in this age group representing two lineages (CC315 and CC395), while the same lineages expressed other serogroup 6 serotypes in children. Taken together, these findings suggest a potential cross-protection of PCVs against serotype 6C IPD among vaccinated children but not among adults. Serotype 6A became the most important serogroup 6 serotype in children but it decreased in adult IPD. No other serogroup 6 serotypes were detected, so available phenotypic or simple genotypic assays remain adequate for distinguishing serotypes within serogroup 6 isolates.

## Introduction

Although historically only two serotypes were recognized within serogroup 6 (6A, 6B), five more were recently discovered (6C, 6D, 6F, 6G and 6H) and shown to be the result of alterations in the *wciN* and *wciP* genes [1,2]. An additional serotype “6E” was proposed based on divergent capsular loci (including SNPs and indels), most frequently of serotype 6B isolates. However, the polysaccharide of this putative serotype, henceforth designated 6B-2, was recently shown to be identical of that of isolates with canonical serotype 6B loci (designated 6B-1) [2].

The 7-valent PCV (PCV7), which includes serotype 6B, was introduced in Portugal in 2001. However, PCV7 was not included in the National Immunization Plan so its uptake increased slowly. Previous work from our laboratory identified a period when no PCV7 attributable changes in serotypes causing invasive pneumococcal disease (IPD) occurred (Pre-PCV7, 1999–2002), early-PCV7 (2003–2006) and late-PCV7 (2007–2009) periods [3–8]. By mid-2009 and start of 2010, 10-valent (PCV10) and 13-valent (PCV13) PCVs were introduced replacing PCV7. While PCV10 was used only briefly and does not include additional serogroup 6 serotypes, PCV13 includes serotype 6A. This study aimed to evaluate which serogroup 6 serotypes were causing IPD in Portugal, the genetic lineages associated and their antimicrobial resistance as well as any changes during a period when PCVs were being introduced.

## Materials and methods

### Isolates and serotyping

In 1999–2012, n = 4812 isolates causing IPD (n = 985 in children <18 yrs and n = 3847 in adults ≥18 yrs) were identified and characterized regarding serotype and antimicrobial susceptibility [3–8]. Isolates were serotyped by the standard capsular reaction test (Statens Serum Institut, Copenhagen, Denmark). Only isolates expressing serogroup 6 (n = 242, 5%) were retained for further study. To confirm the phenotypically determined serotypes and to identify the most recent serotypes and locus class, three PCR reactions were used (Table 1), complemented by sequencing of *wciN* and *wciP*.

**Table 1:** PCRs used for serotype and class identification.

Purpose of the PCR	Target gene	Primer	Sequence (5’-3’)	Target serotype	Product size	Reference
Multiplex PCR for identification of 6A, 6B, 6C and 6D serotypes	*wzy*	wzy-f	CGACGTAACAAAGAACTAGGTGCTGAAAC	Serogroup 6	220 bp	[9]
		wzy-r	AAGTATATAACCACGCTGTAAAACTCTGAC			
	*wciP*	wciP-f1	ATATGTAGAAGAACTGGCTCAGGGTAG	6A, 6C and 6F	128 bp	This study
		wciP-r1	GATGACTAGATGGTACATTATGTCCAT			
	*wciN*	wciN-f1	CATTTTAGTGAAGTTGGCGGTGGAGTT	6C and 6D	727 bp	This study
		wciN-r1	AGCTTCGAAGCCCATACTCTTCAATTA			
Class identification	*wzh*	wzh-f	TGATATTCATTCGCACATTGTC	Class 2 sequences	578 bp	[10]
		wzh-r	TATGAACCAAATCACGCTCCAAG			
	*wze*	wze-f	CTCACAGGCAAAATTGGATTC	Class 1 sequences	217 bp	[10]
		wze-r	AACAGAATTGCGAATATCTC			
*wciN* sequencing	*wciN*	wciN-f2	TGGAAAGATATTGAAATTTT	Serogroup 6	1.4 kb (6A/6B-1/ 6F/6G)	This study
					1.2 kb (6C/6D)	
		wciN-r2	GTT TTTCTTTCAATATCTTTA		1.7 kb (6B-2)	
*wciP* sequencing	*wciP*	wciP-f2	CGATTAATTTTTTATTAATG	Serogroup 6	1.0 kb	This study
		wciP-r2	ATATGAATAAGAAATTTAAAAG			

### Multilocus Sequence Typing (MLST)

MLST was performed as previously described [11]. Briefly, seven housekeeping genes were sequenced and compared to the pneumococcal MLST database (http://pubmlst.org/spneumoniae) to identify the alleles and respective sequence types (STs). PHYLOViZ [12] was used to define clonal complexes (CCs) at double locus variant (DLV) level using only the STs found in this study.

### Antimicrobial susceptibility testing

Etest strips (AB Biodisk, Solna, Sweden) were used to determine the minimal inhibitory concentration (MIC) for penicillin, cefotaxime and levofloxacin. Susceptibility to erythromycin, clindamycin, vancomycin, linezolid, trimethoprim-sulfamethoxazole, tetracycline and chloramphenicol was tested by the Kirby-Bauer disk diffusion technique according to CLSI procedures. Unless otherwise stated, the interpretative criteria prior to 2008 [13] were used. These criteria were selected to enable comparison with previous studies. Multidrug resistance (MDR) was defined by non-susceptibility to at least three classes of antibiotics.

### Statistical analysis

Genetic diversity was evaluated using Simpson’s index of diversity (SID) and respective 95% confidence intervals (CI_95%_) [14]. The Cochran-Armitage test (CA) was used to evaluate the temporal trends of serotypes, STs and CCs. Only STs and CCs with ≥5 isolates were considered for the CA analysis. Fisher’s exact test (FET) was used to test association of serotypes, STs and CCs with age group. The false discovery rate (FDR) correction for multiple testing [15] was used in both tests. A p<0.05 was considered significant for all tests.

## Results and discussion

**Fig 1:**
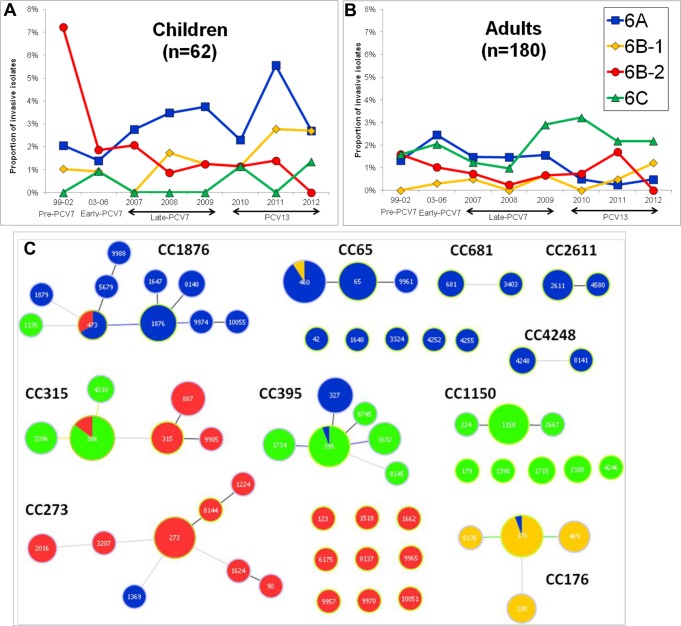
Clonal composition and changes in serogroup 6 serotypes among invasive pneumococci recovered in Portugal (1999–2012). (A) Shows the variations of the serotypes and serotype classes in children and (B) in adults. The years before PCV7 introduction (1999–2002) and subsequent periods were defined as described in the text. (C) shows STs and CCs identified colored by serotype. Each circle represents an ST and the diameter represents its frequency in a logarithmic scale. Grey lines connect STs that are double-locus variants, while lines of other colors connect STs that are single-locus variants according to the PHYLOViZ tie-break rule reached. STs that are linked belong to the same CC. This data set can be explored online at http://bit.do/PHYLOViZ_sero6.

The serotypes identified were 6A (n = 80), 6B (n = 79) and 6C (n = 83). All class 2 loci were identified among serotype 6B isolates (6B-2, n = 52; 6B-1, n = 27). No representatives of serotypes 6D, 6F, 6G and 6H were found, confirming their rarity in Europe [16,17]. The proportion of serogroup 6 isolates was higher in children than in adults [6.4% and 4.7%, respectively, FET p = 0.032] and differences in serotype distribution were also observed (Fig 1 and S1 and S2 Tables). Most 6C pneumococci were recovered from adults (n = 79/83) resulting in an association with this age group [FET p<0.001, significant after FDR correction]. There was no association between serotype and isolate source. Despite no overall change in frequency of serogroup 6 pneumococci following the introduction of PCVs, there were changes in proportion of serotypes (Fig 1). In the pre-PCV7 period, serotype 6B (6B-2) was the most frequent in contrast to neighboring Spain where 6B-1 predominated [16], but it subsequently decreased in both adults and children, similarly to Spain [supported only in children: CA p<0.001, significant after FDR; CA p = 0.160, in adults]. Class 6B-1 accounted for a small proportion of IPD in both age groups before vaccine introduction and, although there was an increase in the proportion of 6B-1 isolates in both age groups, this was supported only in adults and before FDR correction (CA p = 0.041). So although both 6B-1 and 6B-2 express the same polysaccharide, they showed different dynamics following vaccination. It was hypothesized that the two classes could present differences in capsule expression [2] potentially explaining their contrasting variations seen here. Serotype 6A increased to become the most frequent serotype in children in late-PCV7 and PCV13 periods. In adults, although 6A was an important serotype up to 2009, a decrease was seen afterwards (CA p = 0.006, significant after FDR), coinciding with PCV13 introduction in children (Fig 1B). While the proportion of isolates expressing serotype 6C remained low and stable in children, in adults an increase in serotype 6C was noted after 2008, although not statistically supported (CA p = 0.392), establishing it as the most frequent serotype of serogroup 6 in adults.

All isolates were characterized by multilocus sequence typing (MLST) to complement the information already available [18] and PHYLOViZ was used to define CCs (Fig 1C). Serotype 6A was the most diverse (27 STs and 13 CCs) followed by 6B-2 (21 STs and 12 CCs), 6C (17 STs and 9 CCs) and 6B-1 (5 STs and 2 CCs). Still, the majority of serogroup 6 isolates (n = 210, 87%) were distributed into only 7 genetic lineages, three of which included PMEN clones [19]: CC315 (Poland^6B^315, n = 47), CC395 (Portugal^6A^327, n = 37), CC65 (n = 35), CC176 (n = 26), CC273 (Greece^6B^273 and Spain^6B^90, n = 26), CC1876 (n = 22) and CC1150 (n = 17) (S3 and S4 Tables). Mostly, each ST presented only one serotype, with the exception of STs ST176, ST460, ST473, ST386 and ST395, possibly reflecting capsular switching events (Fig 1C). The 6B-2 ST90 lineage dominant in Asia [10,20] was represented by a single isolate.

The genetic diversity of serogroup 6 isolates recovered from both children and adults was similar when considering both STs and CCs, with all seven major lineages present in both age groups (S3 and S4 Tables). Although CC176 was found more frequently in children (FET p = 0.017, not supported after FDR), no association of particular STs or CCs with age group was observed. While in adults the majority of CC315 and CC395 isolates expressed serotype 6C (n = 28/39 and n = 27/31, respectively), in children these lineages expressed mostly other serotypes: CC315/6B-2 (n = 6/8) and CC395/6A (n = 5/6). Serotype 6C was found in 3.0% of carriage isolates in children [21] but in only 0.4% of IPD (S1 Table). Taken together these observations suggest that children may be particularly protected against serotype 6C IPD, potentially through cross-protection due to the presence of polysaccharides 6A and 6B in PCV13. CC315 increased in adults (CA p = 0.012, significant after FDR). This CC included both 6C and 6B-2 isolates but only the 6C drove the increase (CA p = 0.001). In contrast, and in agreement with the decrease of 6B-2, there was a decrease in the major lineage expressing this class (CC273) in both children and adults (CA p<0.001 and p = 0.002, respectively, both significant after FDR) (S1 Fig and S3 and S4 Tables). Increase of the prevalence of serotype 6C pneumococci was reported in several regions [17,22,23]. However, the genetic lineage that increased was not always CC315. For example, in Southampton, England, the increase of serotype 6C pneumococci in carriage was due to the clonal expansion of CC395 [22]. In Spain, the authors associated the increase of serotype 6C isolates with spread of CC1150 [23], although isolates of CC315 emerged in 2007, coinciding with the data from IPD and carriage in Portugal.

Sequencing of the capsular genes *wciN* and *wciP* identified 5 and 14 alleles, respectively (S5 Table). There was a strong correspondence between the alleles at these loci and serotype and locus type but two isolates of ST460 presented unusual capsular loci. In one isolate a point mutation seems to have occurred in an allele characteristic of serotype 6A switching it to 6B (6B-1). The other possibly resulted from horizontal DNA transfer, presenting a hybrid locus including class 1 sequences associated with 6A and 6B-1 (wciN-1) and class 2 sequences (wciP-8). These observations confirm that serotype switching within serogroup 6 may occur in the wild by both point mutation and recombination.

The antimicrobial susceptibility of the isolates is indicated in Table 2. Serotype 6B-2 presented the highest proportion of multidrug resistant isolates (67%) followed by 6C (36%) (MDR, defined as non-susceptibility to at least 3 antimicrobial classes). The majority of isolates non-susceptible to penicillin (PNSP) [13] expressed low level resistance (MIC = 0.12–1 μg/mL), with the exception of a single 6B-2 isolate that expressed high level resistance (MIC = 2 μg/mL). None of the PNSP isolates was 6B-1. Considering the current CLSI guidelines [24], 6 cerebrospinal fluid isolates (6B-2, n = 3; 6C, n = 2; 6A, n = 1) would be considered resistant and all remaining isolates would be considered fully susceptible to penicillin using the non-meningitis breakpoints. Erythromycin resistance (ERP) was identified in 77 isolates and 53 isolates were simultaneously PNSP and ERP (serotype 6C, n = 29; serotype 6B-2, n = 20; and serotype 6A, n = 4). CC315 and CC273 presented the highest proportions of non-susceptible isolates (Table 2). The proportion of isolates representing these CCs changed over time and this was the basis of changes in resistance within serotypes 6C and 6B-2. Among 6C, the proportions of PNSP (p<0.001), ERP (p = 0.001), clindamycin (p = 0.001), tetracycline (p<0.001) and MDR (p = 0.001) increased (CA, all significant after FDR), changes driven mostly by increases in CC315. In fact, the remaining two CCs representing almost all serotype 6C isolates were CC1150, including mostly PNSP, and CC395 including mostly susceptible isolates. In 6B-2, decreases of ERP (p = 0.014), clindamycin (p = 0.005), tetracycline (p = 0.031) and MDR (p = 0.024) resistance were observed (CA, all significant after FDR correction), reflecting the decrease in CC273.

**Table 2:** Antimicrobial resistance of serogroup 6 isolates responsible for invasive infections in Portugal (1999–2012).

	No. of non-susceptible isolates (%)											
	Serotype				Clonal complex							
Antimicrobial ^a^	6A	6B-1	6B-2	6C	CC315	CC395	CC65	CC273	CC176	CC1876	CC1150	Other^b^
MDR	4 (5.0)	3 (11.1)	35 (67.3)	30 (36.1)	47 (100.0)	0 (0)	1 (2.9)	17 (65.4)	3 (12.0)	1 (4.5)	0 (0)	3 (9.4)
PEN	10 (12.5)	0 (0)	26 (50.0)	42 (50.6)	45 (95.7)	1 (2.7)	2 (5.7)	9 (34.6)	0 (0)	4 (18.2)	13 (76.5)	4 (12.5)
MIC_50_	0.023	0.016	0.047	0.064	0.125	0.016	0.023	0.023	0.016	0.016	0.094	0.023
MIC_90_	0.064	0.032	0.19	0.19	0.19	0.032	0.047	0.38	0.023	0.19	0.125	0.064
ERY	4 (5.0)	9 (33.3)	34 (65.4)	30 (36.1)	47 (100.0)	0 (0)	0 (0)	16 (61.5)	9 (34.6)	3 (13.6)	0 (0)	2 (6.3)
CLI	1 (1.3)	4 (14.8)	33 (63.5)	30 (36.1)	47 (100.0)	0 (0)	0 (0)	16 (61.5)	4 (15.4)	0 (0)	0 (0)	1 (3.1)
LEV	0 (0)	0 (0)	0 (0)	1 (1.2)	1 (2.1)	0 (0)	0 (0)	0 (0)	0 (0)	0 (0)	0 (0)	0 (0)
SXT	10 (12.5)	6 (22.2)	27 (51.9)	3 (3.6)	6 (12.8)	3 (8.1)	4 (11.4)	16 (61.5)	5 (19.2)	1 (4.5)	2 (11.8)	9 (26.1)
TET	5 (6.3)	3 (11.1)	34 (65.4)	32 (38.6)	44 (93.6)	0 (0)	1 (2.9)	21 (80.8)	3 (11.5)	0 (0)	1 (5.9)	4 (12.5)
CHL	1 (1.3)	0 (0)	7 (13.5)	2 (2.4)	2 (4.3)	0 (0)	1 (2.9)	6 (23.1)	0 (0)	0 (0)	0 (0)	1 (3.1)

^a^All isolates were susceptible to cefotaxime, vancomycin and linezolid. MDR: multidrug resistance, PEN: penicillin, MIC: minimum inhibitory concentration, ERY: erythromycin, CLI: clindamycin, LEV: levofloxacin, SXT: trimethoprim-sulfamethoxazole, TET: tetracycline, CHL: chloramphenicol. Isolates presenting PEN MIC≥0.12μg/ml were considered resistant and isolates presenting PEN MIC<0.12μg/ml were considered susceptible.

^b^Other CCs or STs not included in those discriminated. MDR: CC4248 (n = 1), ST1518 (n = 1), ST1662 (n = 1); PEN: CC4248 (n = 1), ST1518 (n = 1), ST1662 (n = 1), ST4255 (n = 1); ERY: ST1518 (n = 1), ST1662 (n = 1); CLI: ST1662 (n = 1); SXT: CC681 (n = 1), CC4248 (n = 1), ST42 (n = 1), ST1518 (n = 1), ST1662 (n = 1), ST3324 (n = 1), ST9957 (n = 1), ST9965 (n = 1), ST9970 (n = 1); TET: CC4248 (n = 2), ST1715 (n = 1), ST3324 (n = 1); CHL: ST1662 (n = 1).

The rarity of serotypes 6D, 6F, 6G and 6H indicates that currently available phenotypic or simple genotypic assays remain adequate for distinguishing serotypes within serogroup 6 isolates. Although the 6B-2 class seems to have been preferentially affected by vaccination in Portugal, this was in contrast to what was documented elsewhere [20]. Further studies are necessary to clarify potential differences between the two classes and their response to vaccination. Expansion of resistant 6C lineages in adults, but not in children where the same lineages express other serogroup 6 serotypes, may be a hallmark of the post-PCV period and suggest that vaccination and antimicrobial use are the two major forces currently shaping pneumococcal populations.

## Supporting information

S1 Fig Temporal changes of the proportion of CC273 and CC315 among all invasive pneumococci in Portugal (1999–2012). (PDF)

S1 Table No. of isolates of each serotype of serogroup 6 responsible for invasive infections in children (<18 years) in Portugal (1999–2012). (PDF)

S2 Table No. of isolates of each serotype of serogroup 6 responsible for invasive infections in adults (≥18 years) in Portugal (1999–2012). (PDF)

S3 Table No. of isolates of STs and CCs of serogroup 6 responsible for invasive infections in children (<18 years) in Portugal (1999–2012). (PDF)

S4 Table No. of isolates of STs and CCs of serogroup 6 responsible for invasive infections in adults (≥18 years) in Portugal (1999–2012). (PDF)

S5 Table Allelic profiles of genes *wciN* and *wciP* and respective serotype and CC/ST. (PDF)
