# Supplementary material for: Clonal and serotype dynamics of serogroup 6 isolates causing invasive pneumococcal disease in Portugal: 1999-2012
Source: PLoS One. 2017 Feb 2;12(2):e0170354. doi: 10.1371/journal.pone.0170354 (PMC5289433; doi:10.1371/journal.pone.0170354)
Supplement: S1 Fig — (PDF) [file pone.0170354.s001.pdf]

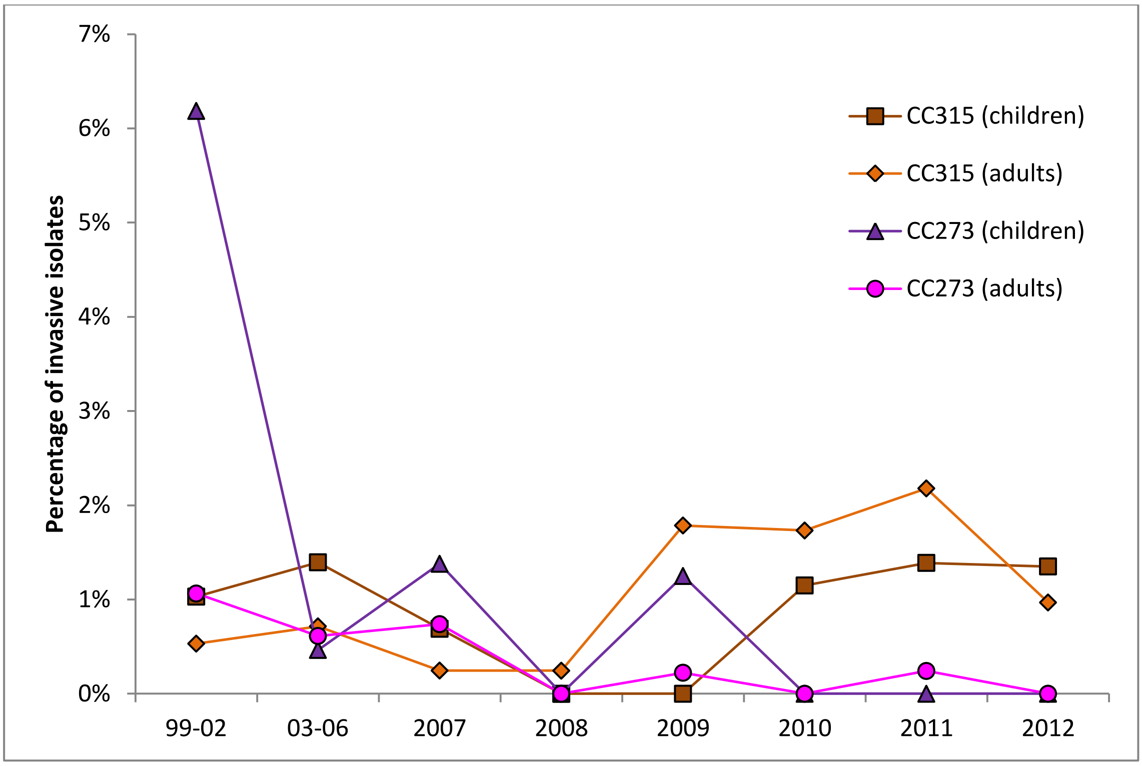

Figure S1. Temporal changes of the proportion of CC273 and CC315 among all invasive pneumococci in Portugal (1999-2012).
