# Supplementary material for: Clonal and serotype dynamics of serogroup 6 isolates causing invasive pneumococcal disease in Portugal: 1999-2012
Source: PLoS One. 2017 Feb 2;12(2):e0170354. doi: 10.1371/journal.pone.0170354 (PMC5289433; doi:10.1371/journal.pone.0170354)
Supplement: S1 Table — (PDF) [file pone.0170354.s002.pdf]

Supplemental Table S1. No. of isolates of each serotype of serogroup 6 responsible for invasive infections in children (<18 years) in Portugal (1999-2012).

	**Pre-vaccine**				**PCV7**							**PCV13**			
	**1999**	**2000**	**2001**	**2002**	**2003**	**2004**	**2005**	**2006**	**2007**	**2008**	**2009**	**2010**	**2011**	**2012**	**Total**
Serogroup 6	2	3	3	2	3	1	2	5	7	7	10	5	7	5	**62**
6A	0	1	0	1	2	0	1	0	4	4	6	2	4	2	**27**
6B-1	0	0	0	1	0	1	0	1	0	2	2	1	2	2	**12**
6B-2	2	2	3	0	1	0	1	2	3	1	2	1	1	0	**19**
6C	0	0	0	0	0	0	0	2	0	0	0	1	0	1	**4**
**All invasive pneumococci**	**20**	**19**	**31**	**27**	**29**	**42**	**51**	**93**	**145**	**115**	**160**	**87**	**72**	**74**	**965**
