# Supplementary material for: Clonal and serotype dynamics of serogroup 6 isolates causing invasive pneumococcal disease in Portugal: 1999-2012
Source: PLoS One. 2017 Feb 2;12(2):e0170354. doi: 10.1371/journal.pone.0170354 (PMC5289433; doi:10.1371/journal.pone.0170354)
Supplement: S2 Table — (PDF) [file pone.0170354.s003.pdf]

Supplemental Table S2. No. of isolates of each serotype of serogroup 6 responsible for invasive infections in adults (≥18 years) in Portugal (1999-2012).

	Pre-vaccine				PCV7							PCV13			
	**1999**	**2000**	**2001**	**2002**	**2003**	**2004**	**2005**	**2006**	**2007**	**2008**	**2009**	**2010**	**2011**	**2012**	**Total**
Serogroup 6	4	1	6	6	13	10	17	17	16	11	26	18	19	16	**180**
6A	0	0	3	2	6	4	6	8	6	6	7	2	1	2	**53**
6B-1	0	0	0	0	1	0	0	2	2	0	3	0	2	5	**15**
6B-2	0	0	3	3	2	3	2	3	3	1	3	3	7	0	**33**
6C	4	1	0	1	4	3	9	4	5	4	13	13	9	9	**79**
**All invasive pneumococci**	**67**	**91**	**113**	**105**	**167**	**214**	**312**	**285**	**406**	**409**	**448**	**404**	**413**	**413**	**3847**
