# Supplementary material for: Clonal and serotype dynamics of serogroup 6 isolates causing invasive pneumococcal disease in Portugal: 1999-2012
Source: PLoS One. 2017 Feb 2;12(2):e0170354. doi: 10.1371/journal.pone.0170354 (PMC5289433; doi:10.1371/journal.pone.0170354)
Supplement: S3 Table — (PDF) [file pone.0170354.s004.pdf]

Supplemental Table S3. No. of isolates of STs and CCs of serogroup 6 responsible for invasive infections in children (<18 years) in Portugal (1999-2012).

	**Pre-vaccine**				**PCV7**							**PCV13**			
	**1999**	**2000**	**2001**	**2002**	**2003**	**2004**	**2005**	**2006**	**2007**	**2008**	**2009**	**2010**	**2011**	**2012**	**Total**
**CC176**	**-**	**-**	**-**	**1**	**-**	**1**	**-**	**1**	**-**	**2**	**2**	**1**	**2**	**2**	**12**
ST176	-	-	-	1	-	1	-	-	-	1	2	1	-	1	7
ST138	-	-	-	-	-	-	-	1	-	-	-	-	1	-	2
ST469	-	-	-	-	-	-	-	-	-	-	-	-	1	1	2
ST8136	-	-	-	-	-	-	-	-	-	1	-	-	-	-	1
**CC65**	**-**	**-**	**-**	**1**	**-**	**-**	**-**	**-**	**2**	**2**	**2**	**2**	**1**	**1**	**11**
ST460	-	-	-	1	-	-	-	-	1	-	2	1	-	1	6
ST65	-	-	-	-	-	-	-	-	1	2	-	1	1	-	5
**CC273**	**2**	**1**	**3**	**-**	**-**	**-**	**-**	**1**	**2**	**-**	**2**	**-**	**-**	**-**	**11**
ST273	1	-	3	-	-	-	-	-	1	-	1	-	-	-	6
ST2016	-	-	-	-	-	-	-	1	1	-	1	-	-	-	3
ST90	1	-	-	-	-	-	-	-	-	-	-	-	-	-	1
ST1224	-	1	-	-	-	-	-	-	-	-	-	-	-	-	1
**CC315**	**-**	**1**	**-**	**-**	**1**	**-**	**1**	**1**	**1**	**-**	**-**	**1**	**1**	**1**	**8**
ST386	-	-	-	-	-	-	-	1	1	-	-	-	-	1	3
ST315	-	-	-	-	1	-	1	-	-	-	-	-	-	-	2
ST887	-	1	-	-	-	-	-	-	-	-	-	-	1	-	2
ST9985	-	-	-	-	-	-	-	-	-	-	-	1	-	-	1
**CC1876**	**-**	**-**	**-**	**-**	**-**	**-**	**-**	**-**	**2**	**2**	**-**	**-**	**3**	**-**	**7**
ST1876	-	-	-	-	-	-	-	-	2	2	-	-	-	-	4
ST473	-	-	-	-	-	-	-	-	-	-	-	-	1	-	1
ST5679	-	-	-	-	-	-	-	-	-	-	-	-	1	-	1
ST9988	-	-	-	-	-	-	-	-	-	-	-	-	1	-	1
**CC395**	**-**	**-**	**-**	**-**	**1**	**-**	**1**	**1**	**-**	**-**	**3**	**-**	**-**	**-**	**6**
ST327	-	-	-	-	1	-	-	-	-	-	3	-	-	-	4
ST395	-	-	-	-	-	-	1	1	-	-	-	-	-	-	2
**CC681**	**-**	**-**	**-**	**-**	**-**	**-**	**-**	**-**	**-**	**-**	**1**	**-**	**-**	**-**	**1**
ST3403	-	-	-	-	-	-	-	-	-	-	1	-	-	-	1
**CC1150**	**-**	**-**	**-**	**-**	**-**	**-**	**-**	**-**	**-**	**-**	**-**	**1**	**-**	**-**	**1**
ST1150	-	-	-	-	-	-	-	-	-	-	-	1	-	-	1
**CC4248**	**-**	**1**	**-**	**-**	**-**	**-**	**-**	**-**	**-**	**-**	**-**	**-**	**-**	**-**	**1**
ST4248	-	1	-	-	-	-	-	-	-	-	-	-	-	-	1
ST1648	-	-	-	-	1	-	-	-	-	-	-	-	-	-	1
ST1662	-	-	-	-	-	-	-	1	-	-	-	-	-	-	1
ST3324	-	-	-	-	-	-	-	-	-	-	-	-	-	1	1
ST8137	-	-	-	-	-	-	-	-	-	1	-	-	-	-	1
