# Supplementary material for: Clonal and serotype dynamics of serogroup 6 isolates causing invasive pneumococcal disease in Portugal: 1999-2012
Source: PLoS One. 2017 Feb 2;12(2):e0170354. doi: 10.1371/journal.pone.0170354 (PMC5289433; doi:10.1371/journal.pone.0170354)
Supplement: S4 Table — (PDF) [file pone.0170354.s005.pdf]

Supplemental Table S4. No. of isolates of STs and CCs of serogroup 6 responsible for invasive infections in adults (≥18 years) in Portugal (1999-2012).

	**Pre-vaccine**				**PCV7**							**PCV13**			
	**1999**	**2000**	**2001**	**2002**	**2003**	**2004**	**2005**	**2006**	**2007**	**2008**	**2009**	**2010**	**2011**	**2012**	**Total**
**CC315**	**-**	**-**	**1**	**1**	**-**	**2**	**3**	**2**	**1**	**1**	**8**	**7**	**9**	**4**	**39**
ST386	-	-	-	-	-	2	1	1	1	1	4	6	5	3	24
ST887	-	-	1	1	-	-	1	-	-	-	-	1	1	-	5
ST3396	-	-	-	-	-	-	-	-	-	-	3	-	1	1	5
ST315	-	-	-	-	-	-	1	1	-	-	-	-	1	-	3
ST4310	-	-	-	-	-	-	-	-	-	-	1	-	1	-	2
**CC395**	**1**	**-**	**-**	**3**	**2**	**3**	**5**	**2**	**3**	**-**	**3**	**5**	**3**	**1**	**31**
ST395	1	-	-	1	2	2	2	-	-	-	2	2	3	-	15
ST1692	-	-	-	-	-	-	-	2	-	-	1	2	-	-	5
ST1714	-	-	-	-	-	-	2	-	2	-	-	-	-	1	5
ST327	-	-	-	2	-	1	-	-	-	-	-	1	-	-	4
ST8145	-	-	-	-	-	-	1	-	-	-	-	-	-	-	1
ST8745	-	-	-	-	-	-	-	-	1	-	-	-	-	-	1
**CC65**	**-**	**-**	**-**	**-**	**2**	**2**	**3**	**3**	**4**	**3**	**2**	**1**	**1**	**3**	**24**
ST460	-	-	-	-	2	2	3	1	3	2	-	-	-	3	16
ST65	-	-	-	-	-	-	-	2	1	-	2	1	1	-	7
ST9961	-	-	-	-	-	-	-	-	-	1	-	-	-	-	1
**CC1150**	**1**	**-**	**-**	**-**	**1**	**-**	**2**	**1**	**1**	**2**	**2**	**2**	**1**	**3**	**16**
ST1150	1	-	-	-	1	-	2	1	1	1	2	2	1	2	14
ST224	-	-	-	-	-	-	-	-	-	-	-	-	-	1	1
ST2667	-	-	-	-	-	-	-	-	-	1	-	-	-	-	1
**CC273**	**-**	**-**	**3**	**1**	**2**	**2**	**-**	**2**	**3**	**-**	**1**	**-**	**1**	**-**	**15**
ST273	-	-	2	1	1	2	-	1	3	-	-	-	1	-	11
ST1369	-	-	1	-	-	-	-	-	-	-	-	-	-	-	1
ST1624	-	-	-	-	1	-	-	-	-	-	-	-	-	-	1
ST3207	-	-	-	-	-	-	-	-	-	-	1	-	-	-	1
ST8144	-	-	-	-	-	-	-	1	-	-	-	-	-	-	1
**CC1876**	**-**	**-**	**1**	**-**	**3**	**-**	**1**	**3**	**-**	**2**	**3**	**-**	**-**	**2**	**15**
ST1876	-	-	1	-	1	-	-	2	-	1	1	-	-	-	6
ST473	-	-	-	-	-	-	-	-	-	1	1	-	-	-	2
ST8140	-	-	-	-	1	-	1	-	-	-	-	-	-	-	2
ST1135	-	-	-	-	-	-	-	-	-	-	-	-	-	1	1
ST1647	-	-	-	-	1	-	-	-	-	-	-	-	-	-	1
ST1879	-	-	-	-	-	-	-	1	-	-	-	-	-	-	1
ST9974	-	-	-	-	-	-	-	-	-	-	-	-	-	1	1
ST10055	-	-	-	-	-	-	-	-	-	-	1	-	-	-	1
**CC176**	**-**	**-**	**-**	**-**	**1**	**-**	**-**	**2**	**2**	**-**	**4**	**-**	**2**	**3**	**14**
ST176	-	-	-	-	1	-	-	2	1	-	3	-	2	2	11
ST469	-	-	-	-	-	-	-	-	1	-	-	-	-	1	2
ST138	-	-	-	-	-	-	-	-	-	-	1	-	-	-	1
**CC2611**	**-**	**-**	**-**	**-**	**1**	**1**	**1**	**1**	**-**	**-**	**1**	**-**	**-**	**-**	**5**
ST2611	-	-	-	-	1	1	1	-	-	-	1	-	-	-	4
ST4580	-	-	-	-	-	-	-	1	-	-	-	-	-	-	1
**CC681**	**-**	**-**	**-**	**-**	**-**	**-**	**-**	**1**	**-**	**-**	**1**	**-**	**-**	**-**	**2**
ST681	-	-	-	-	-	-	-	1	-	-	1	-	-	-	2
**CC4248**	**-**	**-**	**1**	**-**	**-**	**-**	**1**	**-**	**-**	**-**	**-**	**-**	**-**	**-**	**2**
ST4248	-	-	1	-	-	-	-	-	-	-	-	-	-	-	1
ST8141	-	-	-	-	-	-	1	-	-	-	-	-	-	-	1
ST1715	-	-	-	-	-	-	1	-	-	-	-	1	-	-	2
ST2185	1	-	-	-	1	-	-	-	-	-	-	-	-	-	2
ST42	-	-	-	-	-	-	-	-	-	1	-	-	-	-	1
ST123	-	-	-	1	-	-	-	-	-	-	-	-	-	-	1
ST179	-	1	-	-	-	-	-	-	-	-	-	-	-	-	1
ST1390	-	-	-	-	-	-	-	-	-	1	-	-	-	-	1
ST1518	-	-	-	-	-	-	-	-	-	-	-	-	1	-	1

	Pre-vaccine				PCV7							PCV13			
	**1999**	**2000**	**2001**	**2002**	**2003**	**2004**	**2005**	**2006**	**2007**	**2008**	**2009**	**2010**	**2011**	**2012**	**Total**
ST4246	1	-	-	-	-	-	-	-	-	-	-	-	-	-	1
ST4252	-	-	-	-	-	-	-	-	1	-	-	-	-	-	1
ST4255	-	-	-	-	-	-	-	-	1	-	-	-	-	-	1
ST6175	-	-	-	-	-	-	-	-	-	1	-	-	-	-	1
ST9957	-	-	-	-	-	-	-	-	-	-	-	1	-	-	1
ST9965	-	-	-	-	-	-	-	-	-	-	-	-	1	-	1
ST9970	-	-	-	-	-	-	-	-	-	-	-	1	-	-	1
ST10051	-	-	-	-	-	-	-	-	-	-	1	-	-	-	1
