# Supplementary material for: Clonal and serotype dynamics of serogroup 6 isolates causing invasive pneumococcal disease in Portugal: 1999-2012
Source: PLoS One. 2017 Feb 2;12(2):e0170354. doi: 10.1371/journal.pone.0170354 (PMC5289433; doi:10.1371/journal.pone.0170354)
Supplement: S5 Table — (PDF) [file pone.0170354.s006.pdf]

Table S5. Allelic profiles of genes *wciN* and *wciP* and respective serotype and CC/ST.

Allelic profile		Serotype				Clonal complex								
wciN[a]	***wciP***[b]	**6A**	**6B-1**	**6B-2**	**6C**	**CC315**	**CC395**	**CC65**	**CC273**	**CC176**	**CC1876**	**CC1150**	**CC2611**	**Other**[c]
1	1	33	-	-	-	-	-	32	-	-	-	-	-	1
4	1	1	-	-	-	-	-	1	-	-	-	-	-	-
1	2	24	-	-	-	-	-	-	-	1	15	-	-	8
3	2	14	-	-	-	-	8	-	-	-	-	-	5	1
5	2	1	-	-	-	-	1	-	-	-	-	-	-	-
1	4	-	25	-	-	-	-	-	-	25	-	-	-	-
1	8	-	1	-	-	-	-	1	-	-	-	-	-	-
2	8	-	-	51	-	17	-	-	25	-	1	-	-	8
wciN$_\beta$	9	-	-	-	60	28	27	-	-	-	1	-	-	4
wciN$_\beta$	13	-	-	-	18	-	-	-	-	-	-	16	-	2
1	14	5	-	-	-	-	-	-	1	-	4	-	-	-
1	22	1	-	-	-	-	-	-	-	-	1	-	-	-
1	23	1	-	-	-	-	-	-	-	-	-	-	-	1
1	24	-	1	-	-	-	-	1	-	-	-	-	-	-
wciN$_\beta$	25	-	-	-	1	-	1	-	-	-	-	-	-	-
wciN$_\beta$	26	-	-	-	1	1	-	-	-	-	-	-	-	-
wciN$_\beta$	27	-	-	-	1	-	-	-	-	-	-	-	-	1
2	28	-	-	1	-	-	-	-	-	-	-	-	-	1

[a]Since the changes leading to the expression of serotypes 6F e 6G were identified in *wciNα* variants, only these were sequenced for the identification of allelic variants. *wciN$_\beta$* variants detected by PCR (all found in serotype 6C isolates) are indicated as such in the table. The sequence of each allele is available at https://dx.doi.org/10.6084/m9.figshare.3437429.v1.

[b]In two isolates serotype 6C it was not possible to determine the allele of the *wciP* gene. The sequence of each allele is available at https://dx.doi.org/10.6084/m9.figshare.3437429.v1.

[c]Other CCs or STs not included in those discriminated. These were (*wciN-wciP*) – (1-1): ST4255, n=1; (1-2): CC681, n=3; CC4248, n=3; ST3324, n=1; ST4252, n=1; (3-2): ST42, n=1; (2-8): ST123, n=1; ST1518, n=1; ST1662, n=1; ST8137, n=1; ST9957, n=1; ST9965, n=1; ST9970, n=1; ST10051, n=1; (wciN$_\beta$-9): ST1715, n=2; ST179, n=1; ST4246, n=1; (wciN$_\beta$-13): ST2185, n=2; (1-23): ST1648, n=1; (wciN$_\beta$-27): ST1390, n=1; (2-28): ST6175, n=1.
